# Alterations in the Th2 Profile in a Pediatric Population Exposed to PM Emitted by Agricultural and Industrial Activities: A Cross-Sectional Study

**DOI:** 10.3390/toxics14050384

**Published:** 2026-04-30

**Authors:** María Fernanda Romo-García, Oliver Mendoza-Cano, Mónica Ríos-Silva, Jaime Alberto Bricio-Barrios, Pedro Rincón-Avalos, Herguin B. Cuevas-Arellano, Miguel A. Martínez-Preciado, Ángel Gabriel Hilerio-López, Mario López-Rojas, Juan Manuel Uribe-Ramos, Agustín Lugo-Radillo, Yolitzy Cárdenas, Efrén Murillo-Zamora, Juan José Oropeza-Valdez, Mariana Haydee García-Hernández, Bruno Rivas Santiago, Irma Elizabeth González-Curiel, Rafael Julio Macedo-Barragán

**Affiliations:** 1 Laboratorio de Inmunotoxicología, Unidad Académica de Ciencias Químicas, Campus UAZ Siglo XXI, Universidad Autónoma de Zacatecas, Carretera Zacatecas-Guadalajara KM.6, Ejido La Escondida, Zacatecas C.P. 98160, Zacatecas, Mexico; mromog@cinvestav.mx; 2 Facultad de Ingeniería Civil, Universidad de Colima, Carretera Colima-Coquimatlán Km. 9, Col. Jardines del Llano, Coquimatlán C.P. 28400, Colima, Mexico; oliver@ucol.mx (O.M.-C.); princon0@ucol.mx (P.R.-A.); mariolopez@ucol.mx (M.L.-R.); jmuriber@ucol.mx (J.M.U.-R.); 3 Facultad de Medicina, Universidad de Colima, Av. Universidad 333, Las Víboras, Colima C.P. 28040, Colima, Mexico; mrios@ucol.mx (M.R.-S.); jbricio@ucol.mx (J.A.B.-B.); 4 Facultad de Ciencias, Universidad de Colima, Bernal Díaz del Castillo No. 340, Col. Villas San Sebastián, Colima C.P. 28045, Colima, Mexico; hcuevas@ucol.mx; 5 Comisión Nacional del Agua, Dirección Local Colima, Avenida Carlos de la Madrid Béjar S/N, Colonia Centro, Colima C.P. 28000, Colima, Mexico; miguel.martinezpr@conagua.gob.mx; 6 Facultad de Enfermería, Universidad de Colima, Avenida Universidad 333, Las Víboras, Colima C.P. 28040, Colima, Mexico; ahilerito@ucol.mx; 7 Secretaría de Ciencia, Humanidades, Tecnología e Innovación, Facultad de Medicina y Cirugía, Universidad Autónoma Benito Juárez de Oaxaca, Oaxaca C.P. 68020, Oaxaca, Mexico; alugora@secihti.mx; 8 Centro Universitario de Investigaciones Biomédicas, Universidad de Colima, Colima C.P. 28045, Colima, Mexico; rosa_cardenas@ucol.mx; 9 Unidad de Investigaciones en Epidemiología Clínica, Instituto Mexicano del Seguro Social, Av. Lapislazuli 250, Col. El Haya, Villa de Alvarez C.P. 28984, Colima, Mexico; efren-murilloza@imss.gob.mx; 10 Centro de Ciencias de la Complejidad, Universidad Nacional Autónoma de México (UNAM), Mario de La Cueva 20, Coyoacán C.P. 04510, Mexico City, Mexico; juan.oropeza@c3.unam.mx; 11 Unidad de Investigación Biomédica de Zacatecas, Instituto Mexicano del Seguro Social, Interior de la Alameda No. 45, Colonia Centro, Zacatecas C.P. 98000, Zacatecas, Mexico; mariana.haydee.gh@gmail.com (M.H.G.-H.); rondo_vm@yahoo.com (B.R.S.); 12 Facultad de Ciencias Biológicas y Agropecuarias, Universidad de Colima, Tecomán C.P. 28930, Colima, Mexico

**Keywords:** Th2, adaptative immunity TSLP, effect biomarkers, particulate matter

## Abstract

Air pollution with fine particulates is a recent global concern as it has been identified as a risk factor for several health problems. The molecular mechanism linking PM exposure to adverse health effects has not yet been fully elucidated but was found to be linked with inflammatory responses across many investigated exposure settings. In particular, children and juveniles turned out to be highly vulnerable in this regard, with exposure potentially leading to long-term effects on development. We therefore investigated how air pollution in an area where local exposure is traditionally linked with sugar cane production, a potential source of high PM-levels, is affecting the immunologic profile of a pediatric population. Samples were collected from pediatric populations residing in various study sites, including sites with identified sources of high PM emission like sugar cane harvest and thermoelectrical activities and sites with no identified emission sources as control non-exposed population. Significantly elevated TSLP concentrations were observed in serum samples of the pediatric population exposed to particulate matter, along with a distinctive cytokine profile characterized by increased rations of IL-2, IL-4, INF-γ, and IL-6, suggesting alterations in Th2 response. In the present study, no chemical characterization of PM was carried out; however, it was observed by air quality monitoring PM_2.5_ concentrations exceeded permissible limits, suggesting potential exposure of the pediatric population to PM from agricultural activities. These findings underscore the intricate relationship between environmental factors and immune response in vulnerable pediatric populations and explore TSLP response to PM exposition.

## 1. Introduction

Particulate matter (PM) pollution has emerged as a significant public health concern. Exposure to PM has been associated with an increased risk of adverse health outcomes, including a risk factor for chronic diseases such as diabetes. Although the molecular mechanism linking PM exposure to these health effects has not yet been fully elucidated, the proposed pathways share the common feature of inducing chronic inflammation, particularly to environment pollutants like PM [1].

The principal markers of the immunologic response are cytokines. Thymic stromal lymphopoietin (TSLP) is a pleiotropic cytokine, meaning this molecule acts upon multiple cell types, including T and B cells, neutrophils, eosinophils, and innate lymphoid cells. TSLP can act directly on T cells, promoting their proliferation through TCR activation and further amplifying the production of Th2 cytokine by mast and natural killer cells. TSLP plays a critical role in driving Th2-mediated inflammation [1]. TSLP is upregulated by the pro-inflammatory Th2-type cytokines IL-4, TNFα, IL-1β and IL-25, meanwhile interferon IFN-γ and IL-17 negatively regulate TSLP expression [2]. This function of TSLP was initially described by the Ziegler group in transgenic mice, where TSLP induced aerial pathway inflammation characterized by eosinophilic infiltration and Th2 cells, while knockout mice for (TSLPR) did not develop allergic reactions [3] based on these antecedents TSLP has been correlated with asthma episodes in patients.

However, due to its pleiotropic activity, not all TSLP responses have been described, and TSLP may have many other regulatory functions beyond allergic reactions. Recently, it has been reported that TSLP expressions are not only triggered by allergens, but it has also been demonstrated that this molecule can be induced by volatile and non-volatile xenobiotics. For example, in vitro studies have shown that exposure to xenobiotics affects TSLP production and exposure to toluene (50 μM) increases TSLP concentrations compared with controls [4]. Similarly, increased TSLP concentrations have been observed in airway epithelia exposed to fire smoke [5] and structural cells of the nasal mucosa have shown increased levels of IL-6, IL-25, IL-33, and TSLP in response to PM_2.5_ exposure [6].

Despite this evidence, few studies have been conducted in humans, and there is still no consensus regarding normal TSLP concentrations. Characterizing the cytokine profile, including TSLP, in populations exposed mainly to PM could offer advantages for integrative health monitoring because, unlike other effect biomarkers (e.g., trans, trans-muconic acid associated with volatile xenobiotic exposure), cytokines (including TSLP) can be quantified using simple techniques such as enzyme-linked immunosorbent assay (ELISA), providing a promising avenue for biomonitoring xenobiotic exposure and its potential health effects. The study sites were selected and characterized based on data from the Pollutant Release and Transfer Register (RETC). This dataset enabled investigation of particulate matter (PM) dispersion in the atmosphere over the past 17 years (2004–2024) and documentation of the main emission sources, as well as their characterization.

According to this classification, two sites with registers of high-PM emission sources were identified: Queseria (QU) and Campos (CAM). The identified emission source at QU was a sugar-processing plant. Industrial activity at this processing plant begins with the seasonal harvesting and processing of sugarcane for sugar production, a process known as “zafra”, typically conducted during the dry season. Briefly, harvesting is preceded by field burning. The burned clean cane is then rapidly transported to the processing facility to minimize sucrose degradation, where burned cane is crushed to extract juice. This is followed by evaporation, a process powered by steam fired by sugar cane residuals (bagasse) and fossil fuels. The residues from these processes are released into the atmosphere, contributing to increased PM concentrations.

The identified emission source at CAM was a thermoelectric power plant. Study-site selection considered the economic and industrial activities present at each site, as well as RETC data. This approach enabled classification of populations presumed to be exposed to high-PM emission sources and of sites located farther away from such industrial activities.

The composition of PM is highly heterogeneous, and its chemical profile varies substantially influenced by local emissions and environmental conditions; nevertheless, during sugar cane burning and processing for sugar obtention emissions include CO_2_, CO, NO_2_, unburned hydrocarbons [7], as well phenanthrene and anthracene (PAHs) [8]. Specifically, in sugar processing plants composition of PM may include suspended solids, triturated bagasse, organic matter, and other xenobiotics; it has been reported that PM_10_ concentration can reach concentrations up to 159.8 ± 1.4 µg/m^3^ [9]. Regarding the thermoelectric power plant average concentrations range from 10.4 to 39.2 μg/m^3^ composed mainly by PAHs and trace metal elements like Sc, Zn, V, Co, Cr, Pb, Cd, Ni, Cu, Bi, Ge, Ba and Sn [10].

Due to the nature of such compounds, it is feasible to assume that they can be related to respiratory diseases. Some studies relate decreased expiratory flow in children aged 6 to 15 years to an increase of 10 µg/m^3^ PM_10_ and PM_2.5_ [11].

To investigate the effects of exposure to PM on cytokines, an observational cross-sectional epidemiological study was conducted in a pediatric population across seven study sites. These sites comprised both urban and rural areas within the state of Colima.

## 2. Materials and Methods

### 2.1. Quantification of Particulate Matter and Gases Emissions

Particulate matter and gas emissions were quantified with the modular device Nanoenvi (Envira, Asturias, Spain) designed for precise air quality monitoring, utilizing state-of-the-art electrochemical and laser sensors. The device can detect a wide range of pollutants, including carbon monoxide (CO), nitrogen dioxide (NO_2_), ozone (O_3_), PM_10_ and PM_2.5_ and PM_1_ particles, which are key indicators of atmospheric pollution and air quality that significantly impact human health. The device also records essential environmental variables such as temperature, relative humidity, atmospheric pressure, rainfall, solar irradiance, wind speed, and direction, crucial for understanding weather conditions and their influence on pollutant dispersion. For this specific project, the Nanoenvi EQ focused on monitoring a select set of pollutants and environmental parameters: nitrogen oxide (NO), nitrogen dioxide (NO_2_), ozone (O_3_), PM_1_, PM_2.5_, and PM_10_ particles.

### 2.2. Study Design

The study sites were chosen based on their prior classification as high-risk environmental and health sites, determined through simulation studies utilizing RETC data. The sites were classified into those with identified sources of high PM emissions and those without documented sources of PM which were designated as reference sites (Supplementary Table S1).

Queseria (QU) and Campos (CAM) were identified as sites with identified emission sources. QU covers 3 km^2^, and the pediatric population attends schools located 310 m (QU E) and 664 m (QU 20) from the sugar-processing plant, where burned sugarcane is transported seasonally for processing. CAM covers 1.6 km^2^ and is located 6 km from a thermoelectric power plant. This classification primarily relied on the prevalence of industrial, energy-related, or agricultural activities in these regions. The remaining study sites were classified as reference sites due to the lack of identification of high PM emission.

The chosen methodology for the study was an observational cross-sectional epidemiological study. Recruitment of subjects and collection of biological samples took place during the second semester of 2023. The recruitment strategy involved informative sessions with legal guardians of children, where the benefits, risks, and conditions of voluntary participation were communicated. The protocol was authorized by Research Ethics Committee of the Statal Cancer Institute of Colima with the registration number CBCANCL23062023-PRONAII-17. Following the informative sessions, legal guardians, accompanied by their minors, were scheduled to attend sessions in the early morning hours for the signing of informed consent and the assent of the children. Biological samples were then collected from the participants.

Inclusion criteria for the pediatric population comprised an age range from 5 to 12 years and residency in the study sites for at least 5 years. Exclusion criteria included self-reporting of any allergic reactions or consumption of antihistamines, as well as failure to provide informed consent and assent to participate.

This recruitment strategy led to the inclusion of a total of 204 individuals using a non-probabilistic and multistage method. A stratified random sampling procedure, stratified by residential area within the study sites, was employed. The sites were classified into the following groups: the metropolitan area of Colima and Villa de Álvarez (COL), Tecoman (TEC), Armeria (ARM), Manzanillo (MAN), Campos (CAM), Queseria (QU), and Minatitlan (MIN).

### 2.3. Biological Sample Collection

A total of 4 mL of blood was drawn into serum separator tubes to obtain serum samples. Following clot formation, the tubes underwent centrifugation at 2000× *g* for 10 min to separate the serum. The resultant serum was then carefully aliquoted and stored at −20 °C until further analysis. Simultaneously, whole blood samples were collected in EDTA-containing tubes and dispatched to a clinical laboratory for comprehensive hematological analysis.

### 2.4. Cytokine Quantification

IL-2, IL-4, IL-6, IL-10, TNF, IFN-γ, and IL-17A quantification were performed using cytometric bead array cat No. 560484 (Becton Dickinson, San Bernardino, CA, US) the detection limits for cytokine are IL-6 = 2.6, IL-4 = 4.9, IL-6 = 2.4, IL-10 = 4.5, IFN-γ = 3.7, TNF-α = 3.8 and IL-17 = 18.9. Serum samples were incubated with conjugated beads following the manufacturer’s instructions. Subsequently, the samples were analyzed using the FACS Canto II flow cytometer (Becton Dickinson, Franklin Lakes, NJ, USA) and FACSDiva software version 6.1.3 (Becton Dickinson, San Bernardino, CA, USA). TSLP concentration was measured by TSLP Human ELISA Kit cat. No. ab192149 (Abcam, Waltham, MA, USA). Briefly, 50 µL of serum sample were added to ELISA plate together with 50 µL of the Antibody Cocktail and incubated for 1 h. After incubation, plate was washed by triplicate with wash buffer solution and 100 µL of TMB substrate was added and incubated 15 min, posteriorly 100 µL of 0.5 M of sulfuric acid was added to stop the reaction. The protocol was performed according to manufacturer indications and optical density was measured at 450 nm on Multiskan ascent plate reader (Thermo Fisher Scientific, Waltham, MA, USA), the method sensitivity was 3 pg/mL.

### 2.5. Statistical Analysis

First, for the identification of distinctives cytokine profiles of each site, a principal component analysis was performed on Python version 3.12.13 (Supplementary Materials). Differences in TSLP expression and correlation with cytokines on study sites were analyzed by no parametric statistics, Kruskal–Wallis with Dunn’s post-test and Pearson’s correlation respectively. For differences in neutrophilia distribution a proportion analysis was performed by a Yates corrected, Chi-square test. *p* < 0.05 was considered as statistically significant. Unless otherwise stated, all analyses were carried out in GraphPad Prism v5.0 (GraphPad Software, La Jolla, CA, USA).

## 3. Results

### 3.1. Demographic Characteristics

The characteristics of the study population are described in Table 1. Study sites were selected based on the potential exposure to PM according to data obtained from Pollutant Release and Transfer Register (RETC). Queseria site was identified as a population located near a PM emission source due to its proximity to sugar cane-related activities, including processes such as “zafra” (cane combustion). The CAM study site is located 6 km from a thermoelectric plant.

### 3.2. TSLP Concentrations

**Figure 1:**
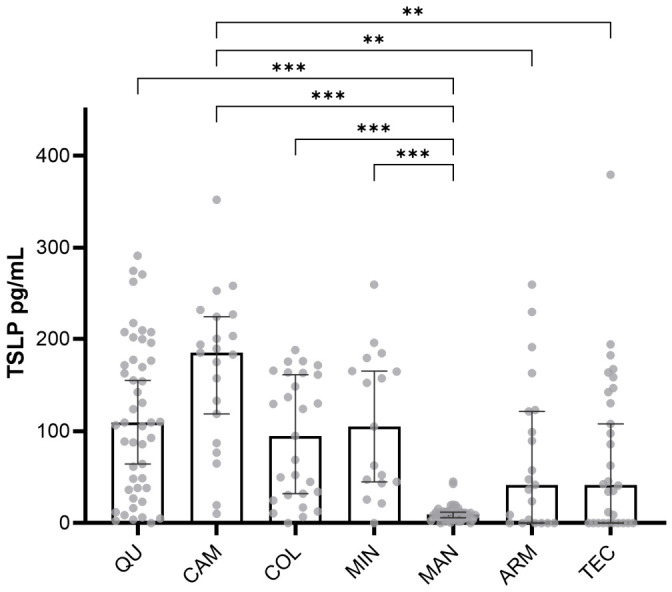
TSLP concentration reported in pg/mL corresponding to the selected study sites. Each subject is represented by a dot in the plot. Median values analyzed by Kruskal–Wallis test with Dunn’s test for multiple comparisons. Error bars are first and third quartiles. ** *p* < 0.01 and *** *p* < 0.001. Abbreviations: COL: Colima—Villa de Alvarez; TEC: Tecoman; ARM: Armeria; MAN: Manzanillo; CAM: Campos; QU: Queseria; MIN: Minatitlán.

To evaluate the response of TSLP to high concentrations of PM, TSLP concentration was measured by ELISA in serum samples in all study sites. TSLP concentrations differed significantly among study sites, with the highest values observed in the pediatric population residing in Campos (CAM) and Queseria (QU) (Figure 1). Based on simulations using RETC data, the potential xenobiotic emission sources in these sites were thermal power generation at CAM and sugar milling/sugarcane production at QU. TSLP concentration shows a wide dispersion of data on QU, thus a stratification of two sampling points within study site was performed for data analysis, the first point was located at 310 mts to the sugar cane mill (QU E) and other at 664 mt (QU 20). When stratified, QU E, the nearest point to the sugar cane mill showed no differences in TSLP expression, probably indicating that the small difference in distance from the emission source was not relevant upon TSLP concentration.

An increase in TSLP alone could suggest an allergic reaction in the evaluated pediatric population (despite the exclusion of self-reported allergic reactions). Allergic reactions correspond to a type I hypersensitivity reaction driven by Th2 response induced by TSLP, a process mediated by effector cells mastocytes, basophils, and eosinophils. To eliminate this condition as the possible inducer of TSLP, the cellular profile of the pediatric population was analyzed. The percentage of basophils was in the range of all subjects of the study sites, while the eosinophiles were out of range of some of them, thus we analyzed differences in eosinophiles (reported as %) among all study as well as differences in proportions of eosinophils out of rage (>5.0% of eosinophiles). No significant differences were observed in eosinophil percentages in CAM residents or in QU (QU 20 or QU E). In the proportion analysis, differences were driven by the MIN site; however, when MIN was excluded, no significant differences were found in the proportions of eosinophils outside the reference range among sites (Table 2). Additionally, to rule out that TSLP concentrations could be increased due to an allergic reaction, a Spearman correlation was performed between eosinophile percentage and TSLP concentrations, no statistical correlation for QU (*p* = 0.78) or CAM (*p* = 0.52) was found. It is therefore unlikely that observed elevated TSLP levels are related to an allergic response in QU (QU 20 or QU E) or CAM population.

### 3.3. Cytokine Profile

**Figure 2:**
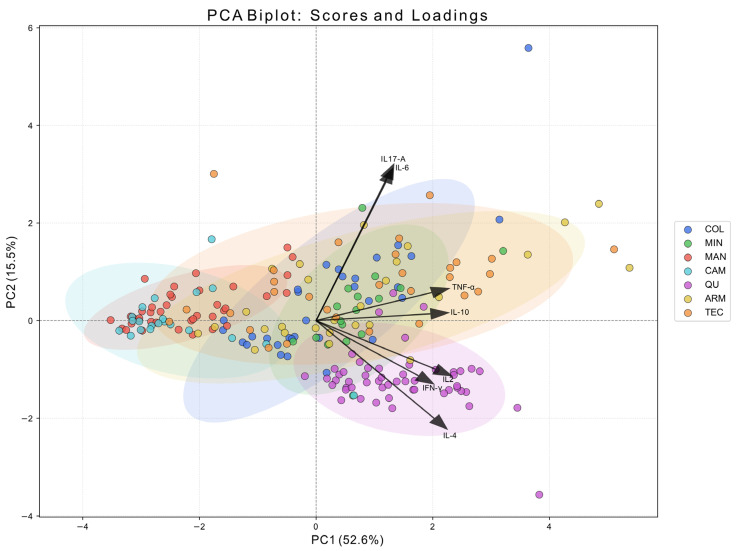
Principal component analysis (PCA) biplot of Th1, Th2, Th17 cytokines. Samples are represented as points and are colored according to their location. Ellipses indicate the dispersion of each group (approximately 95% confidence regions), facilitating visualization of group overlap. Arrows represent the loadings of the measured variables (cytokines), indicating their contribution and direction in the PCA space. All loading vectors originate from the coordinate origin (0,0), as they reflect the projection of each variable onto the principal components. The length and direction of each arrow indicate the strength and orientation of the variable’s contribution to PC1 and PC2, respectively.

TSLP exerts its effects on mast cells and facilitates the differentiation of TCD4 cells into Th2 phenotype by inducing the expression of various interleukins, including IL-4, IL-2, and IL-6. The activity of these interleukins, along with TSLP, is negatively modulated by IFN-γ. Given the interconnected roles of TSLP and interleukins, an investigation was undertaken to assess the cytokine profile in serum samples using flow cytometry (FACS Canto II) with the BD Th1/Th2/Th17 CBA kit. To gain insight into the overall distribution of TH1, Th2, and TH17 cytokine profiles, principal component analysis (PCA) was conducted. Notably, individuals from the QU region exhibited a distinct cytokine profile compared to other study sites, characterized by an upregulation of IL-2, IL-4, and IFN-γ (Figure 2).

**Figure 3:**
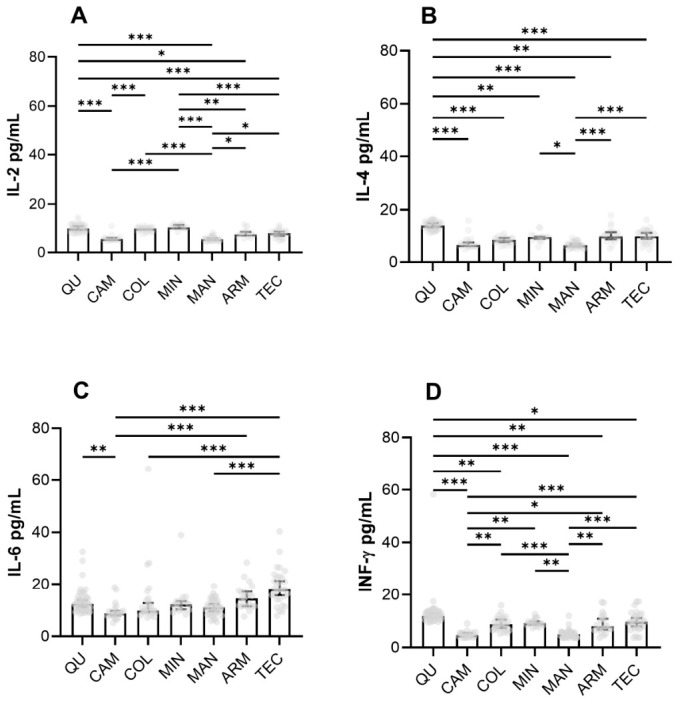
Th2 cytokine concentrations. Pannels show IL-2 (**A**), IL-4 (**B**), IL-6 (**C**) and IFN-γ (**D**) in pg/mL as quantified on serum samples of subjects residing on each study site. Analysis of stratified QU sites near and far from the sugar mill. Each subject is represented by a dot in the plot. Median values were analyzed by Kruskal–Wallis test with Dunn’s test for multiple comparisons. Error bars show the first and third quartiles. * *p* < 0.05, ** *p* < 0.01 and *** *p* < 0.001. Abbreviations: COL, Colima—Villa de Álvarez; TEC, Tecoman; ARM, Armeria; MAN, Manzanillo; CAM, Campos; QU, Queseria; MIN, Minatitlán.

In the QU region, IL-2, IL-4, and IFN-γ were found to be elevated in the cytokine profile of the pediatric population, whereas IL-6 was statistically elevated compared with the CAM population but lower than the ARM and TEC sites (Figure 3).

**Figure 4:**
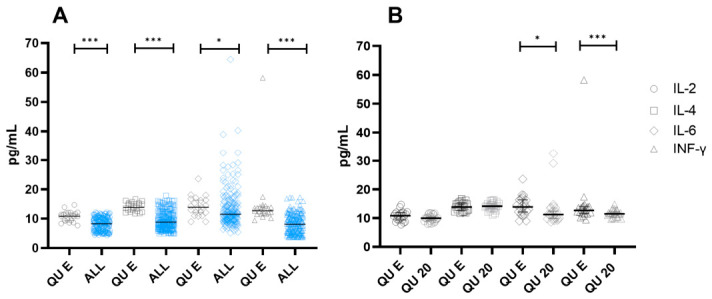
Cytokine concentrations of IL-2, IL-4, IL-6 and IFN-γ. Cytokines were quantified on serum samples and stratified according to residing sites. Panel (**A**) Comparisons of cytokine concentrations at QU (gray color) site against all other sites (blue color). Panel (**B**) Comparisons of cytokine concentrations between QU sites one located nearest (QU E) and farthest (QU 20) from sugar processing plant. In both panels cytokines are represented by the following figures: Circles = IL-2, Squares = IL-4, Rhomboids = IL-6 and Triangles = INF-γ. Each subject is represented by a dot in the plot. Median values were analyzed by Kruskal–Wallis test with Dunn’s test for multiple comparisons. The error bars are first and third quartiles. * *p* < 0.05, and *** *p* < 0.001. Abbreviations: COL: Colima—Villa de Alvarez; TEC: Tecoman; ARM: Armeria; MAN: Manzanillo; CAM: Campos; QU: Queseria; MIN: Minatitlán.

When the data were stratified by points within the QU study site (QU E and QU 20), statistical differences were observed only for IFN-γ and IL-6 withing QU site. However, when QU E point was compared against all study sites (QU 20, ARM, TEC, MAN, MIN, and COL), concentrations of the cytokine profile (IL-2, IL-4, IFN-γ, and IL-6) were higher in QU E, (Figure 4). On contrary to TSLP data, IL-2, IL-4, IFN-γ, and IL-6 seem to be affected by the distance of the emission source. Despite these findings, no increased concentrations of these cytokines were detected in serum samples collected from the CAM study site.

IL-4, IL-2, and IFN-γ are known to regulate TSLP activity. Consequently, we investigated the potential correlation between the elevation of TSLP and the cytokine profile, however, no significant correlation was observed. Nevertheless, a noteworthy correlation was identified between IL-2 and IL-4 in QU E (correlation coefficient: 0.789, *p*-value: <0.001) as well as in QU 20 (correlation coefficient: 0.546, *p*-value: 0.0046). These findings suggest that the increased concentration of specific cytokines observed in the QU study site could indicate a Th2 response to an environmental stressor.

### 3.4. PM Quantification

**Figure 5:**
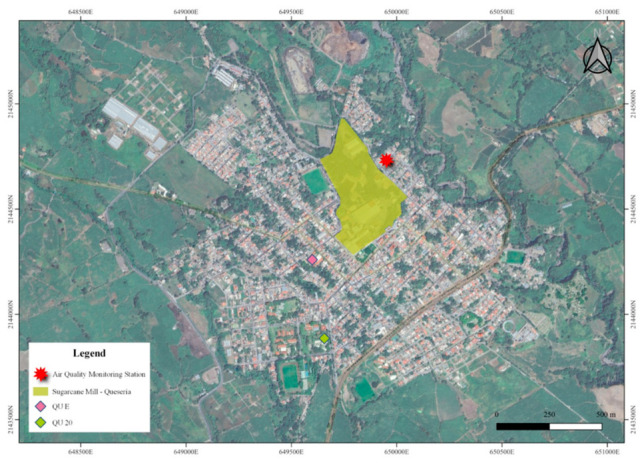
Map of Sugarcane Mill, monitoring station and stratified sampling points (QU E and QU 20) Queseria study site.

Cytokine quantification revealed a characteristic profile, particularly at QU site, one of the sites where an emission source of PM was documented. Based on this observation, our subsequent objective was to verify whether elevated concentration of PM was present at study site. The strategy consisted of air quality monitoring by a station located at 250 mt from the emission source in the QU study site (Figure 5).

**Figure 6:**
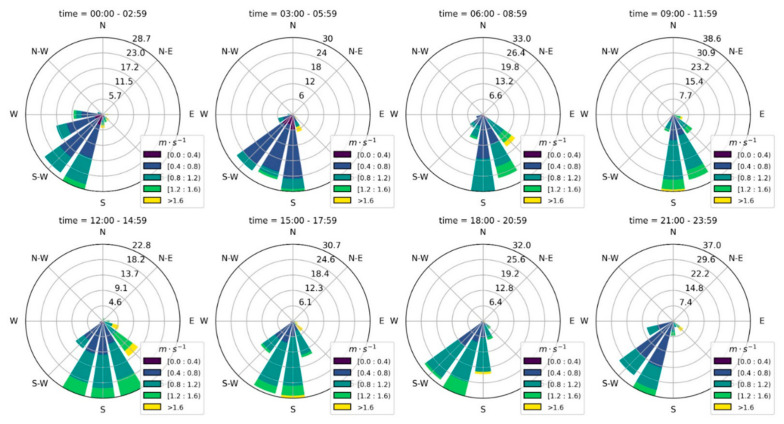
Wind rose (winds speed and direction average) for 2023 by quality monitoring station located on Queseria site.

Data collected from this station revealed the predominant daily airstream patterns. During the monitoring period, it was observed that on some days, the prevailing airstream predominantly flowed in a south-west direction (Figure 6), coinciding with the location where the pediatric population spends a considerable amount of time due to the presence of an elementary school within the study site.

**Figure 7:**
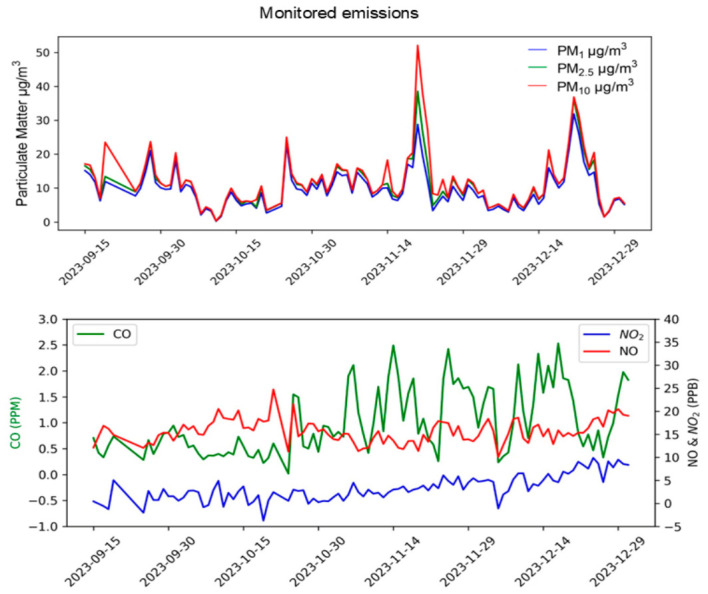
Graph of monitored emissions. Upper Panel: Daily concentrations of PM_1_, PM_2.5_ and PM_10_ in µg/m^3^. Lower panel: Daily concentrations of gases CO reported on in ppm (left y axis), NO and NO_2_ reported in ppb (right y axis). Concentrations measured by air quality monitoring station are graphed against time.

The monitoring station also measured concentrations of particulate matter and gases such as NO and CO. Over a period of four months, the highest detected concentrations were as follows: 21.08 μg/m^3^ for PM1, 23.46 μg/m^3^ for PM_2.5_, 23.70 μg/m^3^ for PM_10_, 20 ppb for NO, 5 ppb for NO_2_, and 1 ppm for CO (Figure 7). Notably, the concentration of PM_2.5_ exceeded the national permissible limits outlined in NOM-021-SSA1-2021 and NOM-172-SEMARNAT-2019 stated at 75 and 45 µg/m^3^ for PM_10_ and PM_2.5_ for acute exposure, these values are considerably higher than the World Health Organization air quality guidelines (PM_10_: 50 µg/m^3^ and PM_2.5_: 25 µg/m^3^). The increase in PM_2.5_ was observed during the month following the sampling period. A monitoring station was situated in CAM site nevertheless maximum PM emission recorded was 19.03 µg/m^3^ for PM_1_, 20.26 µg/m^3^ PM_10_ and 19.94 µg/m^3^ for PM_2.5_, concentrations below the permissible limits (Supplementary Figure S1). The increase in cytokines was statistically significant at the site with the highest concentration of particulate matter (Supplementary Figure S2). This suggests a potentially different TSLP activation pathway in this pediatric population, possibly due to variations in particulate matter emissions from the thermoelectric plant in CAM and the distance of this potential xenobiotic source from residential areas.

## 4. Discussion

According to the database records of RETC, both sites QU and CAM had documented emission sources of particulate matter. However, only at the QU site did quantified PM concentrations exceeded the permissible limits. This finding may support a site-specific association with the cytokine profile observed in that population, which was not replicated at CAM site.

At QU site, the most likely sources of PM emissions are the sugar processing plant (the registered emission source) and the pre-harvest sugarcane burning. The overall process (including sugarcane combustion, transport and industrial processing for sugar production) is associated with PM release.

Pre-harvest burning of sugarcane releases PM into the atmosphere and leaves the soil bare, which causes significant soil dust resuspension. It should be noted that, because the sugarcane processing plant is surrounded by sugarcane fields, PM is likely carried by the wind to the monitoring station regardless of wind strength and direction. Additionally, harvesting occurs during the dry season, when low soil moisture in the bare soil enhances the dispersal and resuspension by gusts and squalls of wind. A previous study showed that sugarcane pre-harvest burning released 60% of PM_2.5_ while resuspended soil dust represented 14% of them. In addition, the contribution of resuspended soil dust and pre-harvest biomass burning to PM_10–2_._5_ represented 51% and 25%, respectively [12].

However, it is feasible that this is not the only source, and some emissions may also be attributable to fuel used in the processing plant. Regarding the energy source, the sugarcane processing plant uses fuel oil and sugarcane bagasse as fuels. In Southeastern Brazil it was documented that oil combustion from sugarcane mills released 12% and 20% of PM_2.5_ and PM_10–2.5_, respectively [12]. The composition of pre-harvest sugarcane emissions factors is widely heterogeneric. These emissions have a mean PM_2.5_ of 2.49 ± 0.66 g kg^−1^ and hazardous polycyclic aromatic hydrocarbons, carbonyls, and volatile organic compounds among which naphthalene, formaldehyde and benzene are the predominant ones, respectively [13]. Although fuels contribute proportionally to PM emissions, most have been reported to originate from bagasse processing for example, in Mexican sugar mills, the burning of sugarcane bagasse for energy production generated in 2015, 104.5 thousand tons of particulates where 96% corresponds to PM_2.5_ [14].

At QU site, air quality parameters measured by the monitoring station during the 2 months surrounding the sampling period were below the permissible limits (CO 11 ppm, PM_10_ 40 µg/m^3^ and NO_2_ 0.210 ppm), only PM_2.5_ concentration was above the permissible limits for PM_2.5_ of 25 µg/m^3^ for acute exposure [15], during the subsequent months (November and December). This increase in PM is consistent with the cane harvest period, with activities concentrated around the sugar-processing plant. Notably, the plant is closer to the QU E point, where IFN-γ and IL-6 were higher than at QU 20, a more distant point from the plant. It must be noted that the site of emission quantification is not located at the investigated residential area and depending on meteorologic conditions, the actual emissions there might even exceed quantified concentrations.

PM_1_ and PM_2.5_ had been associated with respiratory diseases with a 1.09 and 1.06 risk increase per 10 μg/m^3^ rise for PM_1_ and PM_2.5_ respectively, which size is associated with pulmonary but not with upper respiratory tract ailments [16]. Especially, PM_1_ is highly harmful for pediatric population due to its high density and easy deposition and penetration into pulmonary tissues [17]. In children PM_1_ has been associated with short-term increases in asthma; meanwhile, this effect is not observed on adults [18]. The specific vulnerability of the pediatric population to particulate matter is not fully explained, some hypotheses point to differences in the ratio of pulmonary (lung)/body mass meaning that the exposure to same dose of xenobiotic dose by inhalation may be higher in children [19]. Another possible explanation consists of alterations on alveoli formation, a process that occurs during childhood [20].

The cytokines prominently expressed in the cytokine profile of the QU study site (IL-4, IL-2, and IFN-γ) play crucial roles beyond their involvement in the differentiation of naïve T cells into a type two helper (Th2) phenotype. Specifically, this process involves the sequential stimulation of IL-2 and then IL-4, leading to combined phosphorylation of STAT5 and STAT6 [21]. IL4 and IL-2 are associated also to other functions; historically IL-4 has been associated with allergy through its ability to stimulate class switching to IgE [22] and it is also involved in tissue repair and in the regulation of IL-17 mediated inflammatory diseases [23,24], the increase in this cytokine could be related to tissue repair as a response to inhaled particulate matter. IL-4 and IL-2 play essential roles in antibody production by promoting B cell proliferation and preferentially stimulating the production of IgM antibodies over IgE, as noted by Galanaud et al. [25]. However, their primary function lies in enhancing the proliferation of activated CD4+ and CD8+ T cells, thereby augmenting natural killer cell cytotoxicity, as reported by Lehmann et al. [26]. This cellular response is commonly observed following xenobiotic exposure, particularly through interactions with aryl hydrocarbon receptors, as elucidated by Moreno-Nieves et al. [27]. Collectively, these findings suggest a specific Th2 response to particulate matter released during the sugar cane production process.

At the CAM site, an increase in TSLP concentrations was observed, but this increase was not observed in other cytokines. This effect can be related to the presence of PM at low concentrations due to the distance of the documented emission source, which is situated 6 km from the CAM site, whereas the documented emission source in QU is inside the residential area (664 m from one school). Although PM_2.5_ characterized at sugarcane production sites has a similar composition (e.g., Cl, Cu, Zn, Cr, Cd, and Pb) [28,29] a future analysis of the composition of the PM is required.

Also, other possible exposure pathways must be evaluated, since TSLP is not only expressed by airway cells but also in smooth muscle cells, ocular tissues [30], epithelial cells and keratinocytes [31]. PM_2.5_ are a complex mixture, the effects upon human health are difficult to determine even, the exposition to particulate matter could cause other effects non evaluated in this study like cardiovascular disease, disfunction on central nervous system and reproductive alterations [32,33].

Th2 cytokines concentrations were increased in pediatric population of study sites with documented sources of PM identified by RECT, pointing to Th2 as a response to PM exposition on pediatric population. Additionally, average TSLP concentrations among study sites were determined, TSLP of ARM, CAL, COL and MIN were lower compared to QU and CAM sites, these study sites had a median of TSLP concentration of 44.11 pg/mL, near to other basal values reported in pediatric population by other authors (50 pg/mL) [34].

The main activity on QU, sugar cane process could also be an scenario for pesticide exposure nevertheless most of literature support cytokines alteration to PM exposure, for example, in a cohort of 2001 pregnant women with high concentrations of organic pollutants, pesticides, and metals did not present increased odds for elevated concentrations of IgE, TSLP or IL-33 [35]. But there is an association between sugar cane process to adverse pulmonary effects like respiratory distress, acute respiratory illness [36]; in animal models like female BALB/c mice exposed during 4 weeks to biomass-burning-derived PM presented lung impairment and airway inflammation [37]. In this study, a distinctive Th2 cytokine profile in pediatric population residing near to PM emission sources was described, demonstrating immunological alterations, nevertheless deeper research is needed to affirm the exposure to burned cane biomass is the only triggering factor of the alterations on cytokine profile and increase on TSLP concentrations.

This study limitations are the period of air quality monitoring which could not reflect long-term exposure patterns but acutes ones. Also, the absence of PM chemical speciation and the cross-sectional design limits causal inference, restricting the ability to establish temporal relationships between exposure and outcomes.

## 5. Conclusions

Exposure of pediatric populations to PM_2.5_ and other particulate materials is a global concern due to the vulnerability of this age group. The principal aim of this study was to evaluate cytokine alterations in a pediatric population residing in sites with Identified PM emission sources. Particulate matter quantified at the study site by an air quality monitoring station during the two months surrounding the sampling period confirmed that PM_2.5_ concentrations exceeded permissible limits, with increases during the sugarcane harvest season. The pediatric population residing in this site showed a distinct cytokine profile, with increased IL-4 and IL-2, whose primary function is to support proliferation and activation of immune effector cells, a response commonly observed following xenobiotic exposure, suggesting alterations in the Th2 response to high particulate matter concentrations, particularly the PM_2.5_ fraction. Regarding TSLP, concentrations were increased not only in the population exposed to high PM concentrations but also in those in which an emission source had been identified, even when concentrations did not exceed permissible limits. Given that TSLP is an alarmin, these findings may suggest airway damage in the population even at relatively low particulate concentrations.

## Figures and Tables

**Table 1:** Descriptive statistics of study population variables.

	MIN	TEC	ARM	MAN	COL	QU	CAM	*p* Value
	Sites Without Identified PM Emission Sources					Sites with Identified PM Emission Sources		
Number of values	17	29	21	39	30	47	21	
Age (years)								
25% Percentile	7	7	6	7	7	7	6	≤0.05
Median	7	8	7	9	8	9	8	
75% Percentile	8	9	8	10	8	10	9	
Gender								
Male	6	14	9	21	10	26	12	≥0.05
Female	11	15	12	18	20	21	9	

Notes: Table with number of subjects per site and variables. Statistical differences were determined by Kruskal–Wallis with Dunn’s post-test and gender proportions between study sites was determined by a chi square analysis. Abbreviations: COL: Colima—Villa de Álvarez; TEC: Tecomán; ARM: Armería; MAN: Manzanillo; CAM: Campos; QU: Queseria; MIN: Minatitlán. Armería, Colima—Villa de Alvarez and Manzanillo are classified as urban areas.

**Table 2:** Proportion of subjects with anormal and normal percentage of eosinophil.

Site	MIN	TEC	ARM	MAN	COL	QU 20	QUE	CAM	*p* Value
Subjects with eosinophilia	13	17	12	14	7	13	9	8	0.016
Subjects with normal eosinophil values	4	12	9	25	23	11	14	13	
**Site**	**QUE**				**ALL**				***p* value**
Subjects with eosinophilia	9				89				0.511
Subjects with normal eosinophil values	14				103				

Table with number of subjects per site with normal and increased values of eosinophils. Statistical differences for proportions between study sites was determined by a chi square analysis. Abbreviations: COL: Colima—Villa de Alvarez; TEC: Tecoman; ARM: Armeria; MAN: Manzanillo; CAM: Campos; QU E: Queseria, point located at 310 mts to the sugar cane mill); QU 20: Queseria, point located at 664 mts to the sugar cane mill; MIN: Minatitlan.

## Data Availability

The original contributions presented in this study are included in the article/Supplementary Materials. Further inquiries can be directed to the corresponding authors.

## Supplementary Materials

The following supporting information can be downloaded at: https://www.mdpi.com/article/10.3390/toxics14050384/s1, Table S1: Summary of Population, Identified Pollutants and Emission Sources; Figure S1: Daily concentrations of PM1, PM2.5 and PM10 reported in µg/m3. Concentrations measured by air quality monitoring station are graphed against time; Figure S2: Th2 cytokine profile concentrations on serum samples of subjects of stratified sites according to maximum limits of PM2.5 detected.

## Abbreviations

The following abbreviations are used in this manuscript:

TSLP	Thymic Stromal Lymphopoietin
ELISA	Enzyme-linked immunosorbent assay
RETC	Pollutant Release and Transfer Register
PM	Particulate matter
