# Supplementary material for: Alterations in the Th2 Profile in a Pediatric Population Exposed to PM Emitted by Agricultural and Industrial Activities: A Cross-Sectional Study
Source: Toxics. 2026 Apr 30;14(5):384. doi: 10.3390/toxics14050384 (PMC13211662; doi:10.3390/toxics14050384)
Supplement: Supplementary file 1 [file toxics-14-00384-s001.zip › toxics-4237176-supplementary.pdf]

Supplementary Table S1: Summary of population, identified pollutants and emission sources.

Study site	Site	Pollutants	Matrix	Emission Source	Data
Campos (CAM)	Rural	Arsenic, Cadmium, Cyanide, Chromium and Mercury	Air	Electric power generation in a thermoelectric power plant	RETC, 2021
Queseria (QU)	Rural	Arsenic, Nickel Lead and Polycyclic aromatic hydrocarbons	Air	Combustion process in a sugar processing plant	RETC, 2021 and 2016.
Armería (ARM)	Urban	No Data	Not specified	Not specified	RETC, 2021.
Colima and Villa de Alvarez (COL)	Urban	Cadmium, Cyanide, Mercury and Arsenic	Air / Soil	Not specified	RETC, 2021.
Tecoman (TEC)	Rural	Cadmium, Chromium Mercury, Arsenic and Benzene	Not specified	Cement and quicklime processing	RETC, 2021.
		Pesticides	Water / Soil	Agro-industrial sector	
Manzanillo (MAN)	Urban	No Data	Not specified	Not specified	RETC, 2021.
Minatitlan (MIN)	Rural	Chromium, Nickel, Arsenic, Lead and Manganese	Water	Metallurgical (including steelmaking)	RETC, 2021.
		Formaldehyde and Acetaldehyde	Air		

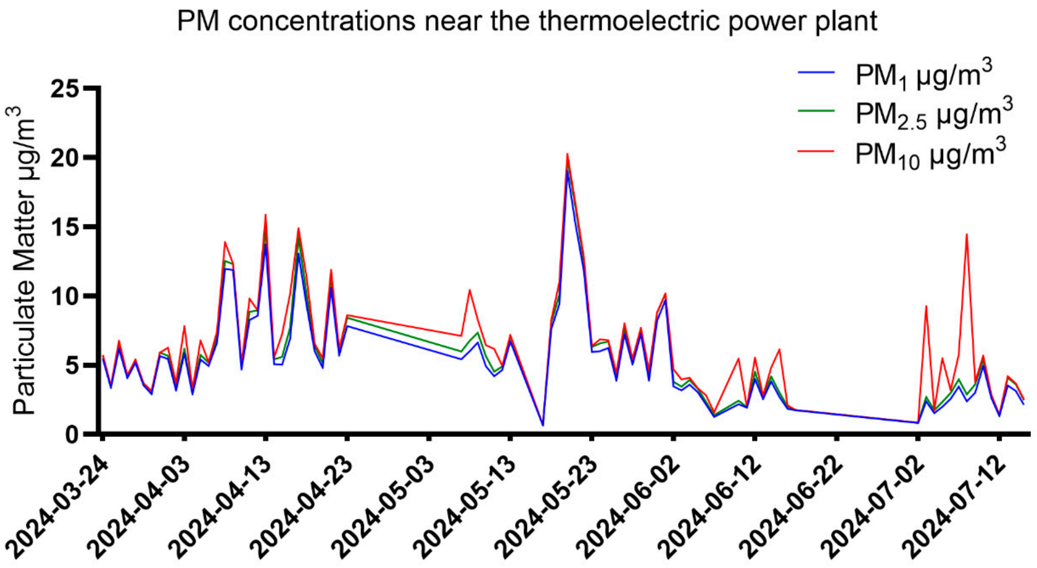

Supplementary Figure S1: Daily concentrations of PM_1, $PM_{2.5}$ and PM_{10} reported in µg/m3. Concentrations measured by air quality monitoring station are graphed against time.

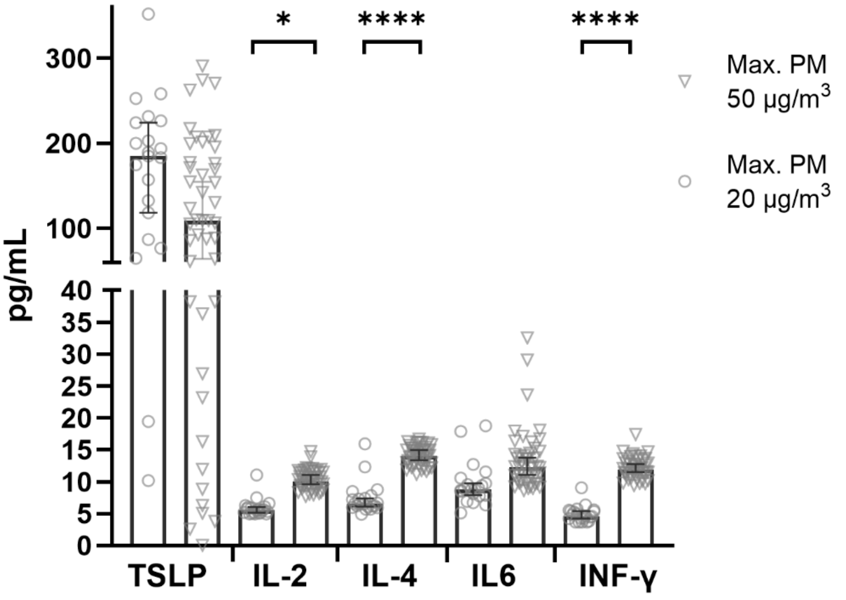

Supplementary Figure S2: Th2 cytokine profile concentrations on serum samples of subjects of stratified sites according to maximum limits of $PM_{2.5}$ detected. Each subject is represented by a dot in the plot. Median values were analyzed by Kruskal-Wallis test with Dunn´s test for multiple comparisons. The error bars are first and third quartiles. *p<0.05, **p<0.01 ***p<0.001 and ****p<0.0001.
